# Regression Modeling and Optimization of CNC Milling Parameters for FDM-Printed TPU 95A Components

**DOI:** 10.3390/mi16101078

**Published:** 2025-09-24

**Authors:** Kaan Emre Engin, Zihni Alp Cevik

**Affiliations:** 1 Department of Mechanical Engineering, Adiyaman University, Adiyaman 02040, Turkey; 2 Department of Electronic and Automation, Besni Ali Erdemoglu Vocational School, Adıyaman University, Adıyaman 02302, Turkey; zcevik@adiyaman.edu.tr

**Keywords:** 3D print, TPU, regression, MRR, surface roughness, Taguchi, machining

## Abstract

Additively manufactured thermoplastic polyurethane (TPU 95A) is widely used in engineering, yet its machining behavior remains insufficiently explored. This study investigates the post-processing machinability of FDM-fabricated TPU 95A using CNC milling, with a particular focus on material removal rate (MRR) and surface roughness (Ra). A full factorial design of experiments (81 runs) is conducted, considering four input parameters such as spindle speed (N; 2000, 4000, 6000 rpm) and feed rate (F; 100, 200, 300 mm/min) on the CNC vertical machining center, together with infill density (ϕ; 33%, 66%, 100%) and layer thickness (LT; 1.0, 1.5, 2.0 mm). MRR is modeled and optimized across all densities, achieving strong fit (R^2^ = 0.94; Adj-R^2^ = 0.93). The optimum conditions are found to be MRR ≈ 1251 mm^3^/min at F = 300 mm/min, ϕ = 100%, N ≈ 3500 rpm and LT ≈ 1.05 mm. Ra can only be measured for 100% infill specimens, as lower infill surfaces violate profile measurement requirements. Its regression model shows weak explanatory power (R^2^ = 0.14; Adj-R^2^ = 0.03) and is excluded from optimization. Instead, Ra is reported descriptively: milling reduced roughness from ≈25–30 μm (as-printed) to ≈13.8 μm under favorable conditions. Overall, the study highlights machining’s role in the hybrid manufacturing practice.

## 1. Introduction

In recent years, additive manufacturing technology has been rapidly advancing. The additive manufacturing process has evolved from equipment used for desktop-sized 3D printing, which includes simpler applications, to industrial-grade additive manufacturing systems [1]. The variety of products that can be manufactured by additive manufacturing methods is wide, such as simple components for prototypes and industrial scale products [2].

Additive manufacturing is an umbrella term. There are many sub-manufacturing categories under additive manufacturing, such as Directed Energy Deposition (DED), Powder Bed Fusion (PBF), VAT Photopolymerization (VPP), Binder Jetting (BJ), Material Jetting (MJ) and Material Extrusion (MEX). Amongst these categories, Extrusion-Based Additive Manufacturing (MEX) is popular and has a wide variety of usages. The popularity comes from the Fusion Deposition Modeling (FDM) method, which is a sub-branch of MEX and has been in the mainstream market for a long time. In the FDM method, material is heated and extruded from a nozzle in multiple layers to produce the desired component [3].

In recent years, advancements in additive manufacturing methods have brought many advantages over conventional subtractive machining methods. Especially in the production of complex shapes, additive manufacturing methods produce parts much more rapidly and provide flexibility in the design and production process [4]. These improvements have expanded the product range that can be produced by utilizing additive manufacturing; however, to obtain the best efficiency, optimization of the process and the materials used in additive manufacturing have become a necessity [5].

Although metallic materials can also be utilized in additive manufacturing, polymeric materials are used in the process to a greater extent than metallic materials. Among many polymeric materials, thermoplastics materials are highly favored and preferred polymeric materials to be used in additive manufacturing applications [2]. Polyurethane thermoplastic (TPU) is one of the thermoplastic materials that can be used in additive manufacturing. TPU combines elastomeric flexibility with sufficient hardness and strength, while also providing abrasion and tear resistance. In addition, TPU is known for its biocompatibility, meaning it does not cause detrimental effects when in contact with biological systems [5]. These advantages, when combined with the ability to create complex shapes in additive manufacturing, make TPU usable in medical applications [6], the textile and footwear sector [7,8] and even in wearable electronics and energy-absorbing applications [9,10].

The internal structure of TPU consists of alternating soft and hard copolymer segments (polyols and diisocyanates). These components cause TPU to have both elastomer and thermoplastic properties. Thus, TPU materials offer both flexibility and strength [11]. Moreover, TPU has high adhesion and compatibility with other polymeric materials that provide the potential to produce multi-material products. TPU can also serve as a matrix material in the production of composite materials. It is possible to obtain various composites due to the use of many composite reinforcement materials with a TPU matrix, such as boron nitride nanosheets (BNN) [12], hybrid hexagonal boron nitride (h-BN) [13], graphene [14] and carbon nanotubes (CNT) [15].

There are many studies on FDM and on the use of TPU. For example, studies have stated that TPU components produced using FDM and powder bed fusion (PBF), a more advanced method, have different mechanical properties and that the material produced with PBF exhibits a more brittle behavior [16]. Most existing studies, however, focus on optimizing printing process parameters to improve the surface, geometric, or mechanical properties of TPU parts.

For instance, the research performed by Liu et al. examines the effect of nozzle temperature and layer thickness on the strength of TPU components [17]. Bruere et al. focus on investigating the temperature range of the nozzle combined with drying conditions to remove moisture from TPU filaments [17,18]. Lin et al. bring attention to the feeding ratio and infill speed parameters to prevent problems like under-extrusion and stringing caused by the low viscosity of TPU [19].

Marco et al. [5] compare infrared light powder bed fusion with FDM systems and show that optimizing layer thickness, line distance and filling speed in open FDM greatly improves mechanical response, with elongation rising to 1162% and elasticity by 45% compared to injection molding. Pelayo et al. [20] identify wall thickness as the key factor that influences the viscoelastic behavior of MEX-printed TPU and based on this, propose a generalized Maxwell model as a viable tool to describe its mechanical response. Xu et al. [21] produced TPU parts using the Selective Lazer Sintering (SLS) method and observe that proper orientation, controlled post-processing and especially the mix ratio of new to reused powders have critical effects on their mechanical properties. Hasdiansah et al. [22] employ the Taguchi method (L27 fractional) and ANOVA analysis to optimize the surface quality of FDM-printed TPU, identifying layer thickness as the most significant factor. The best Ra is achieved at 110% flow rate, 0.1 mm layer thickness, 210 °C nozzle temperature, 30 mm/s print speed, 75% overlap and 100% fan speed.

The inherent material properties like flexibility, low hardness, abrasion resistance and melting temperature of TPU materials are important aspects that contribute to their usage in additive manufacturing applications. However, these same features—especially flexibility and viscoelasticity—also make machining challenging, which is why responses such as Ra and MRR should be studied to understand hybrid workflows. As is evident from the literature, studies mainly focus on the production side of the process and how to improve the outcomes by employing different approaches, largely ignoring post-processing. There is a noticeable lack of studies investigating the subtractive machining processes, such as milling, of the additively manufactured products like TPU and the effect of variable machining parameters on the surface roughness and material removal rate (MRR). This situation indicates a gap of knowledge for those who are seeking to utilize these materials in hybrid manufacturing processes, and requires attention.

Although FDM can produce near net shape parts, some applications require further finishing operations. For TPU, these applications often involve biomedical devices, flexible wearables, or energy-absorbing prototypes, where both dimensional accuracy (MRR) and smooth surfaces (Ra) are essential. These examples include sealing faces that require tight dimensional accuracy, biomedical and wearable device applications where smooth contact surfaces are necessary to prevent irritation on the patients and vibration, or energy absorbing elements where surface uniformity improves bonding or assembly. In such cases, a hybrid path is followed to achieve the required conditions. First, the part is 3D-printed according to the predetermined dimensions, then the critical areas are milled to improve accuracy and surface finish. This motivates the present study, which evaluates how spindle speed, feed rate, infill density and layer thickness affect productivity (MRR) and surface quality (Ra) in post-processed TPU components. The novelty of this study lies in systematically examining the machinability of flexible TPU 95A by integrating CNC milling with FDM-produced parts. The approach combines quantitative optimization of MRR with a descriptive assessment of Ra, demonstrating how milling improves surface finish compared to as-printed parts. In doing so, it fills an important gap in hybrid additive–subtractive manufacturing and provides practical insights that earlier studies have not addressed. Although the present work focuses on TPU 95A, the same experimental and statistical framework can in principle be extended to other FDM-printed polymers with appropriate material-specific considerations.

Based on this motivation, the present study is designed as follows: TPU specimens are fabricated by using FDM and subjected to CNC milling process. Different values of feed rate and spindle speed are used to machine TPU specimens with distinct infill densities and layer thicknesses. Then, surface roughness of the milled specimens and MRR values are measured. As the last step, Taguchi analysis is employed in conjunction with Analysis of Variance (ANOVA) to determine the most effective parameter combinations.

## 2. Materials and Methods

### 2.1. Material and Production

TPU polymeric material is used for experimental work. TPU is generally used for engineering applications, providing both flexibility and hardness at the same time. The manufactured parts can be bent, but exhibit resistance to deformation, and also provide dimensional stability, flowability and an absence of cracking or warping, making them appropriate for additive manufacturing and industrial applications.

TPU 95A filaments indicating a Shore hardness of 95A, with a diameter of 1.75 mm and white in color, which are produced by SAVA, a local filament brand, are used as the sample materials. Mechanical properties of TPU 95A filaments are given in Table 1.

**Figure 1:**
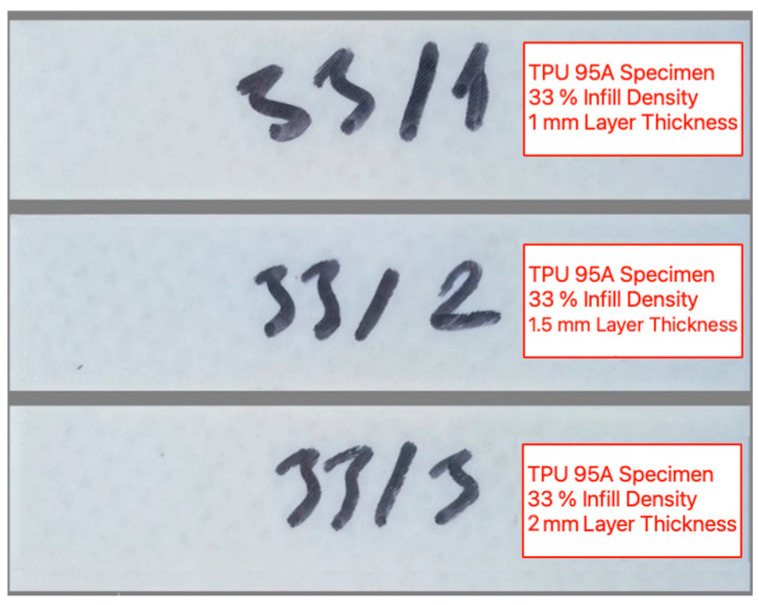
Fabricated TPU 95A specimens with 33% infill density and different layer heights.

An FDM type 3D printer (Bambu X1 Carbon) is employed for the fabrication of TPU 95A specimens. The printer is adjusted to operate at a nozzle temperature of 220 °C, bed temperature of 55 °C, nozzle diameter of 0.4 mm and 3 different layer thicknesses of 1 mm, 1.5 mm and 2 mm. For all prints, the top and bottom layers are set to 1 layer each (equal to LT), with 3 perimeters (shells) and a grid infill pattern at 33%, 66% or 100%. The printing speed is set to 25 mm/s. This printing speed is chosen after preliminary trials and in line with common practice for TPU 95A, as it ensures stable extrusion without deformation or stringing, whereas higher speeds lead to dimensional and bonding problems. All specimens are produced under identical printing settings and from the same filament batch to ensure consistency. Although TPU is hygroscopic, no additional drying step is applied, since all printing is conducted under controlled laboratory conditions (20–25 °C, 40–50% RH) and no moisture-related printing issues are observed. A total of 81 specimens are fabricated with the dimensions of 100 mm × 25 mm × 6 mm. This specimen geometry is deliberately chosen for practical reasons. An overall length of 100 mm provided enough flat surfaces to be milled at the desired cutting parameters. A width of 25 mm matches the CNC vertical machining center’s clamping range. At the same time, the geometry is compact enough to allow efficient fabrication of 81 parts without excessive time or material. An example of the fabricated specimens with 33% infill densities are given in Figure 1.

### 2.2. The CNC Milling Process

The fabricated specimens are subjected to the milling process, using a Mitsubishi MCV-M5 H CNC vertical machining center. Technical specifications of the CNC vertical machining center are given in Table 2.

The milling process of the TPU 95A specimens are performed under 3 different feed rates (F) of 100, 200 and 300 mm/min and spindle speeds (N) of 2000, 4000 and 6000 rpm. Cutting depth is kept constant for all conditions and adjusted to 1 mm. Preliminary trials for deeper cuts (1.5 and 2 mm) lead to vibration, material deformation and degraded surface finish, due to the flexible and viscoelastic nature of TPU 95A. Therefore, a 1 mm cutting depth is selected as the most stable and repeatable value for cutting.

**Figure 2:**
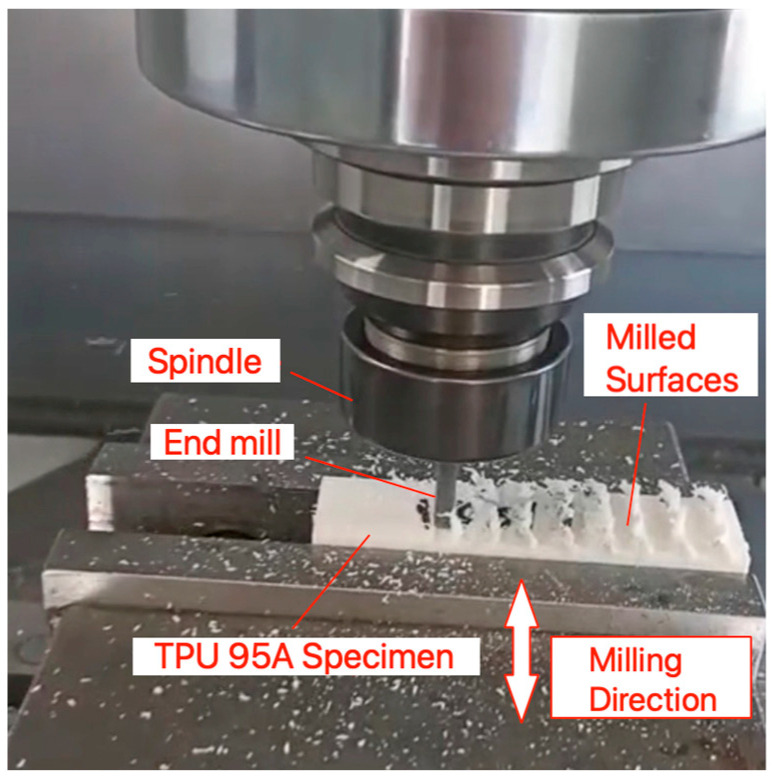
An example on the milling of a TPU specimen.

The cutting tool is an important asset in the milling process. End mill is used to perform the milling process of the fabricated specimens. To prevent dullness, the end mill is replaced with an identical one after every three experiments. This procedure resulted in 27 tool changes across the full 81 runs. The specifications of the end mill are given in Table 3. An example of the TPU 95A materials subjected to milling is given in Figure 2.

The milling process is carried out side by side in the width direction of the parts with the 6 mm diameter end mill and continued along the entire length of the specimen, as demonstrated in Figure 2. A total of 12 channels are milled along the length of the specimen with a step-over value of 8 mm that results in a 2 mm thick residual wall between adjacent channels. The specimens are intentionally left with a 3 mm margin on both sides to allow secure clamping during measurements, to prevent cutting right at the free edges, to reduce the risk of edge defects, tool exit marks and deformation in the soft TPU 95A material.

### 2.3. Surface Roughness and Material Removal Rate (MRR)

Surface roughness is a crucial parameter in every machining process. Additionally, surface roughness of the machined material can be significantly influenced by the infill densities and the flexible nature of additively manufactured TPU 95A materials, making it one of the most critical output parameters in the milling process of TPU materials.

**Figure 3:**
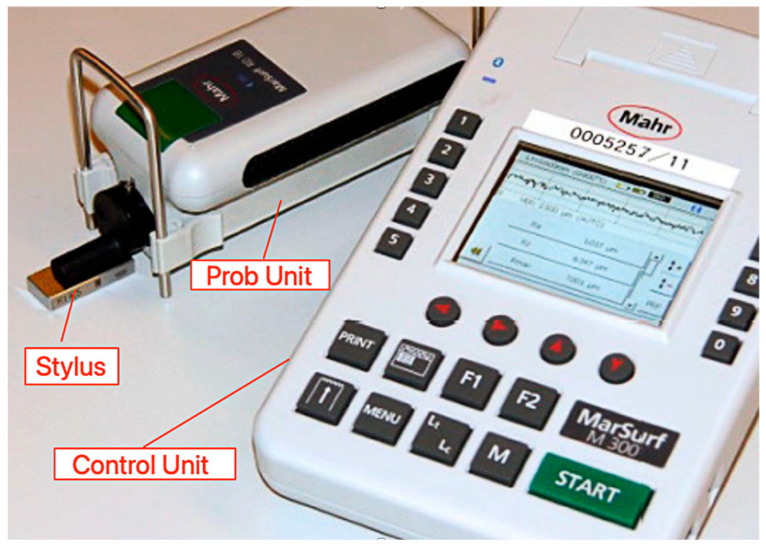
Profilometer (Mahr MarSurf M 300 C) with control unit, probe unit and stylus used for Ra measurements.

Surface roughness values of the milled parts are measured using a Mahr Marsurf m300c-wired mobile profilometer, which is shown in Figure 3.

Surface roughness (Ra) values are measured with a cut-off length λc = 2.5 mm and an evaluation length Le = 12.5 mm, in accordance with ISO 4287/4288. Traces are taken along the channel direction (25 mm width length), centered on the milled floor and kept ≥1.5 mm away from both channel ends and side walls to avoid edge effects. For each specimen, 5 channels are randomly selected and 3 repeated traces per channel are measured, resulting in 15 traces in total. Then, mean Ra is calculated. As-printed Ra on matching top surfaces is measured with the same settings for baseline comparison.

Alternative surface characterization techniques such as 3D optical profilometry or SEM are considered, but these methods do not yield ISO-compliant Ra values and are outside the available laboratory scope. For reliability, each Ra value reported here represents the average of 15 profilometer traces (5 channels × 3 repetitions), which already captures within-specimen variability even though separate error bars are not displayed.

Material removal rate (MRR) is known as the volume of the material removed from the produced material in unit time. The MRR values of the specimens are calculated using Equation (1) as follows:

(1) $$MRR=\frac{V}{T}$$

where V is the volume of the material removed during the machining cycle (mm^3^) and T is the total machining time (min), including both non-cutting and cutting motions. The total volume for a full end milling of a specimen with 100% infill density can be calculated as follows:

(2) $$V_{100}=a_{p}\;a_{e}\;L_{c}^{100}$$

where ap is the depth of cut in the axial direction (mm), ae is the width of cut in the radial direction (mm), and $L_{c}^{100}$ is the total cutting length (mm) for full infill. However, 100% infill density indicates that the component is entirely solid. Additive manufacturing provides the ability to produce parts with lower infill densities. When the infill density is lower than 100%, only a fraction of the entire cutting length is actively involved in material removal. For this reason, the total removed volume during the machining cycle becomes the following:

(3) $$V(\phi )=a_{p}\;a_{e}({\phi L}_{c}^{\left(100\right)})$$

where ϕ accounts for the infill density. The time spent on a total cycle of milling includes the cutting and non-cutting motions. If the feed rate is assumed to be constant for all situations, the total time spent can be calculated as the cutting path length and traverse (indexing) path length and can be written as follows:

(4) $$T\left(\phi \right)=\frac{\phi L_{c}^{100}+L_{i}}{f}$$

where L_i_ is the total traverse (indexing) distance between cuts (mm) and f is the feed rate (mm/min). By substituting Equations (3) and (4) with Equation (1), simplifying can be expressed as follows:

(5) $${MRR}_{corr}\left(\phi \right)=\left(f\;a_{p}\;a_{e}\right)\frac{\phi L_{c}^{100}}{\phi L_{c}^{100}+L_{i}}$$

The nominal MRR, which only accounts for active cutting time, can be expressed in Equation (6) as follows:

(6) $${MRR}_{nom}\left(\phi \right)=f\;a_{p}\;a_{e}\;\phi$$

Consequently, the revised MRR can be expressed as follows:

(7) $${MRR}_{corr}\left(\phi \right)={MRR}_{nom}\left(\phi \right)\frac{L_{c}^{100}}{\phi L_{c}^{100}+L_{i}}$$

Equation (7) shows that the corrected MRR values are less than nominal MRR values, and can be obtained by multiplying the nominal MRR by a duty-cycle correction factor. This factor ($\frac{L_{c}^{100}}{\phi L_{c}^{100}+L_{i}}$), represents the proportion of the total cycle time spent on actual cutting engagement. Even at 100% infill density (ϕ = 1), this coefficient remains less than unity. This is because traverse (indexing) movements do not contribute to volume removal in the milling process; yet, these movements also consumed time and should be included in the total time consumed. At lower infill densities, both nominal and corrected MRR values decrease proportionally due to the volumetric scaling by ϕ, while the ratio between them remains constant under the present definition. This behavior reflects the fixed traverse distance in the toolpath, which does not shorten with reduced material volume. In the present analysis, the infill structure is assumed to be homogeneous, such that the specified infill density represents a uniform volumetric reduction in the solid material throughout the machined region, neglecting local variations in bead geometry or pore distribution. Both nominal values (cutting time only) and corrected values of MRR (cutting + indexing time) for TPU 95A specimens are calculated.

### 2.4. Experimental Design and Statistical Analysis

The Taguchi method minimizes the required experiment number and analyzes the effect of the process parameters on the outcomes statistically. This method determines the proper combinations of control factors by using orthogonal design tables and this increases the efficiency of experimental process [23].

For this study, four process parameters are selected for the milling of TPU specimens. The defined parameters include the spindle speed and feed rate of the machining center, together with the layer thickness and infill density of the fabricated TPU material. There are four control factors, each defined at three levels, and an L9 (3^4^) orthogonal array indicating that just nine experimental runs would have been sufficient to obtain the necessary results. The factors and their corresponding levels are given in Table 4.

However, in this study, a full factorial experimental design consists of 81 experimental runs are deliberately performed rather than following the reduced orthogonal array. The motivation behind this kind of approach is to capture higher-order interactions and nonlinear responses of viscoelastic TPU 95A that may not be visible in a fractional design. Each parameter combination is physically tested once, with no replicates, to ensure complete coverage of the design space. Furthermore, due to the viscoelastic and flexible nature of TPU, specimens have the potential to exhibit nonlinear behavior during milling. For this reason, relying on a reduced number of experimental trials carries the potential to increase the risk of underestimating the effect of noise factors and higher-order interactions. Therefore, a full factorial design is used to make the findings more reliable.

Although a full factorial design is employed, the Taguchi signal-to-noise (S/N) ratios are also calculated. This is not intended as a conventional Taguchi optimization, but only as a supplementary robustness indicator to facilitate comparison with the prior machining literature. The optimization and conclusions in this study are based on regression and ANOVA, with S/N ratios serving only as an auxiliary interpretive tool. In parallel, response optimization is conducted through regression-based modeling in JMP software (V18) using the Fit Model platform. For the analysis, the Standard Least Squares method is used, incorporating the main effects and two-factor interactions (N × F, N × LT, F × LT). Model performance is evaluated by using analysis of variance (ANOVA), coefficients of determination (R^2^, adjusted R^2^) and root mean square error (RMSE). Model adequacy is confirmed by verifying ANOVA assumptions through formal residual diagnostics. The Shapiro–Wilk test (W = 0.881, *p* < 0.0001) indicated a deviation from strict normality. However, residual–predicted and Q–Q plots (Supplementary Figures S1 and S2) showed approximate symmetry and constant variance, supporting the adequacy of the regression models for practical interpretation.

Since the aim is to cover full factorial, each of 81 parameter combinations are tested once and without any replicates. For Ra, multiple profilometer traces (5 channels × 3 repetitions per channel) are averaged per specimen, while MRR values are deterministically derived from toolpath geometry and CNC cycle times. For this reason, error bars or confidence intervals are not included. In the case of MRR, variability is essentially negligible, since values come directly from toolpath geometry and verified cycle times. In the case of Ra, averaging across multiple profilometer traces already captures measurement variation.

## 3. Results

The input process parameters and measurement results are given in Table 5. The MRR values for both nominal and corrected values are calculated. When the full cycle, which includes both cutting and indexing time, is taken into consideration, MRR_corr_ values are lower than nominal values for all feed rates and infill densities. This outcome arises from the inclusion of indexing motions that prolong overall process time without contributing to volume removal. Across all feed rates at all infill densities, there is approximately 23% reduction in MRR values, showing that the MRR_corr_ values are about 77% of the nominal value for all feed rates. This constant ratio results from the application of Equation (6), where the infill density (ϕ) is added during the calculation of MRR_nom_, while the duty-cycle factor remains independent of ϕ. During the experimental phase of the study, the milling operation is performed with both cutting length and indexing length fixed. As a result of this operational condition, reduced infill density does not shorten the indexing distance but only scales the actual material volume along the cutting path. Consequently, lower infill densities proportionally reduce values via the volumetric term, while the corrected/nominal ratio remains unchanged. MRR_corr_ values are used in all procedures of the study. The regression results unsurprisingly yield a perfect fit (R^2^ = 1.00) because the MRR values are derived from deterministic toolpath equations. To ensure that this outcome is not only theoretical, the MRR_corr_ values are compared with machining cycle times automatically logged by the CNC controller. The measured and calculated values agreed within ~1%, confirming that the correction factor and the equations reliably capture actual machine behavior.

Surface roughness (Ra) values can only be measured from specimens with 100% infill density and cannot be measured from specimens with 33% and 66% infill densities. A 100% infill density corresponds to a fully solid specimen, whereas specimens with lower infill densities exhibit discontinuous surfaces with voids. This situation does not satisfy the profile-based roughness measurement requirement which should be performed on a continuous and a stationary surface profile. Partial infill presents open pores, wall and void transitions and local deformations after the milling process that dramatically interrupt the measurement length. Moreover, the viscoelastic nature of TPU 95A amplifies form defects and waviness on porous surfaces, leading to biased Ra values that do not represent the true surface micro-texture. For these reasons, Ra values are reported only for 100% infill surfaces, and not for partial infill density surfaces, because it is not possible to obtain reliable and comparable measurements. Nevertheless, this limitation does not undermine the study’s main purpose. Specimens with low infill densities are included because their MRR behavior is industrially relevant. Many printed TPU components and their applications, such as lightweight lattices, biomedical parts and energy absorbers, are post-processed for dimensional accuracy, even though Ra cannot be meaningfully quantified.

When the surface roughness results (Ra) are examined in Table 5, it is observed that spindle speed, feed rate and layer thickness have effect on the Ra values, but their effects do not follow a linear manner. The lowest Ra values do not occur at a fixed setting of any single parameter. Instead, the lowest Ra values emerged only at specific combinations of feed rate, layer thickness and spindle speed. For example, parameter configurations that can yield the lowest Ra value for a specific layer thickness prove to be disadvantageous in another thickness value. For a specific parameter setting where N = 2000 rpm and F = 200 mm/min, the surface roughness value increases from Ra = 13.82 µm to Ra = 22.94 µm when layer thickness changes from LT = 1.0 mm to LT = 1.5 mm, before decreasing again to Ra = 17.66 µm at LT = 2.0 mm. This situation clearly shows that a parameter combination yielding a better surface quality at one layer thickness can result in lower surface quality at another, highlighting the effect of interactions between parameters. This nonlinear pattern and parameter interactions make it difficult to tune the parameters in a straightforward fashion. This nonlinear behavior mainly comes from the viscoelastic nature of TPU 95A. After the tool passes, the material tends to spring back slightly, which creates small surface waviness. Because TPU is more flexible than rigid plastics, it can also deform locally during cutting. In addition, its tendency to stick to the tool surface sometimes causes irregular textures. These combined effects explain why Ra values do not follow a simple linear trend and why the smoothest surfaces only appear at certain parameter combinations.

However, since Ra values can only be measured from TPU 95 A specimens with 100% infill densities, modeling Ra values for other infill densities is impractical. This situation limits any joint MRR–Ra multi-response optimization due to incomplete data. A reliable multi-response optimization can only be performed when each response is observed completely across the same experimental matrix [24,25]. Therefore, in this study, optimization is restricted to MRR across all densities, while Ra results are used only descriptively to illustrate milling-related improvements compared to as-printed surfaces. This approach prevents misinterpretation and ensures that optimization focuses only on robustly modeled responses (MRR).

For the analysis of 100% infill specimens, model summary statistics are given in Table 6. ANOVA, parameter estimates and effect test results are given in Table 7, Table 8 and Table 9, respectively.

The regression analysis of MRR exhibits a perfect fit for 100% infill density (R^2^ = 1.00; Adj-R^2^ = 1.00; RMSE = 0.0026 mm^3^/min; *n* = 27). ANOVA and effect tests show that feed rate is the most significant parameter (*p* < 0.0001), whereas layer thickness and spindle speed are not significant (both *p* = 1.00). The parameter estimates confirm a strong linear scaling of MRR with feed rate, consistent with the volumetric definition of material removal, while other listed factors remain neutral within the tested ranges.

**Figure 4:**
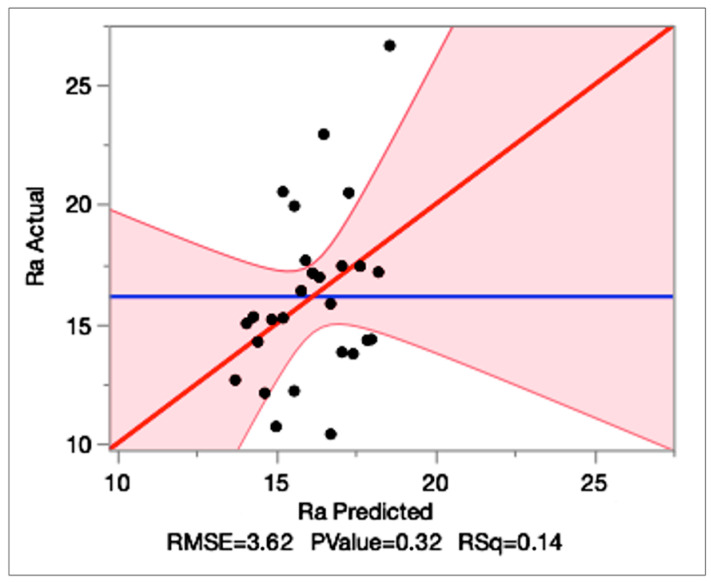
The actual Ra vs. predicted Ra plot graph.

**Figure 5:**
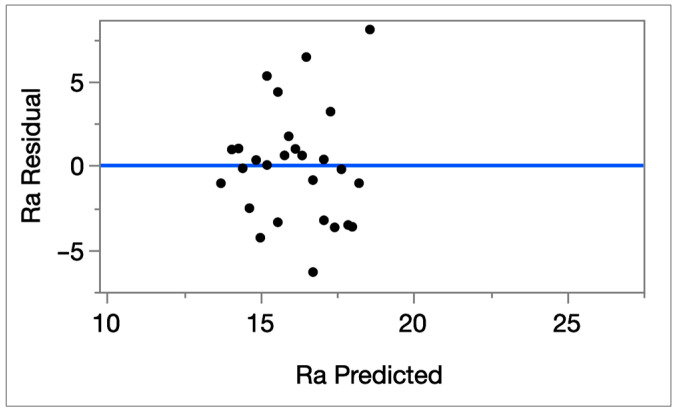
The Ra residual vs. Ra predicted plot graph.

In contrast, it is observed that the Ra regression model exhibits low explanatory power (R^2^ = 0.140; Adj-R^2^ = 0.027; RMSE = 3.62 μm; *n* = 27) and the overall model is also not significant (*p* = 0.32), as can be seen in Table 7. None of the main parameters reaches 5% significance except feed rate, which only shows a near-significant trend as can be seen in Table 9 (*p* = 0.09). The actual vs. predicted Ra plot shows a weak alignment, which is illustrated in Figure 4, and the residual by predicted plot indicates large unexplained variance, which supports the earlier observation that minimum Ra occurs at specific conditions and not at a single fixed level of any factor. The residual by predicted plot is given in Figure 5. These plots demonstrate the poor fit of the Ra model, justifying its exclusion from optimization.

On the other hand, it is important to note that milling of TPU materials continues to deliver a noticeable improvement: the Ra of as-printed TPU surfaces is typically around ≈25 µm and ≈30 µm, while the lowest Ra value obtained after milling is ≈13.8 µm. This confirms the benefit of post-processing, even though Ra could not be reliably modeled.

In line with this separation, a dedicated optimization of MRR is conducted for all infill density levels (33%, 66% and 100%). The objective is to maximize productivity. The optimization is performed in JMP, using the desirability function framework. MRR is defined as a “larger-is-better” response. Each factor is evaluated not only for its individual contribution but also for its interactions within this framework. This situation ensures that optimization captures the true sensitivity of the process. By extending optimization to the entire dataset, the analysis can identify the optimal conditions for achieving maximum MRR at different infill densities of TPU 95A specimens. In this way, optimization can become valid even in the absence of surface roughness data.

The regression analysis results are given in Table 10. The regression analysis confirms the robustness of the model, with R^2^ = 0.94 and Adj-R^2^ = 0.93. This value indicates that over 93% of the variance in MRR is explained by the selected parameters. RMSE is found to be 92.52 mm^3^/min against a mean response of 645.67 mm^3^/min, based on 81 observations. These values show accuracy and stability in predictions. ANOVA results are given in Table 11.

ANOVA results in Table 11 confirm the overall significance of the regression model (*p* < 0.0001). This value validates that chosen process parameters have a strong influence on MRR. The F-ratio has a high value of 221.51 which indicates that the proportion of variance in MRR explained by the model substantially exceeds the residual variance, thus demonstrating that the regression model is statistically robust and suitable for further optimization.

Following the confirmation of overall model significance, the parameter estimates are analyzed, and the results are given in Table 12.

Parameter estimates given in Table 12 show the individual effects of process factors on MRR. Feed rate has a significant positive coefficient (*p* < 0.0001), while spindle speed and layer thickness are statistically insignificant (both *p* = 1.0000). On the other hand, layer thickness affects the printed specimen geometry but does not alter the programmed engagement parameters of the milling toolpath.

In comparison, infill density is observed to have effects on MRR values. Specimens with 33% and 66% infill densities display reductions of −248.87 mm^3^/min and −33.30 mm^3^/min, respectively, relative to the 100% infill baseline (*p* < 0.0001 and *p* = 0.0248). In the model, 100% infill is used as the baseline level. The coefficients show that specimens with 33% and 66% infill achieve significantly lower MRR compared to the solid specimens, highlighting that maximum productivity is obtained only at full infill.

This situation is consistent with previous studies in additive manufacturing, where higher infill densities improve the effective material removal during the machining of the parts and contribute to the structural integrity of the fabricated materials [26,27]. An additively manufactured material with higher infill density offers a greater material volume to be cut along the programmed toolpath. A denser infill increases the fraction of the cycle, during which the tool removes material and elevates the time-averaged MRR under identical conditions. As the infill density decreases (ϕ < 1), due to the absence of material, the proportion of the toolpath engaged in cutting becomes smaller, while the non-cutting indexing distance remains unchanged. This reduction lowers the MRR values. This means that specimens with partial infill have lower cycle-averaged MRR than fully dense specimens.

The results of the effect test are given in Table 13. It is observed that the value of each factor strengthens the findings. Feed rate again emerges as the dominant parameter (F ≈ 657.5, *p* < 0.0001), with infill density ranking second (F ≈ 225.0, *p* < 0.0001). In contrast, layer thickness and spindle speed show no significant influence (F ≈ 0, *p* = 1.0000). This confirms their negligible role in material removal efficiency. These findings are consistent with earlier studies, which have also shown that MRR is governed mainly by feed rate and material engagement volume, rather than spindle speed or layer thickness [28,29].

**Figure 6:**
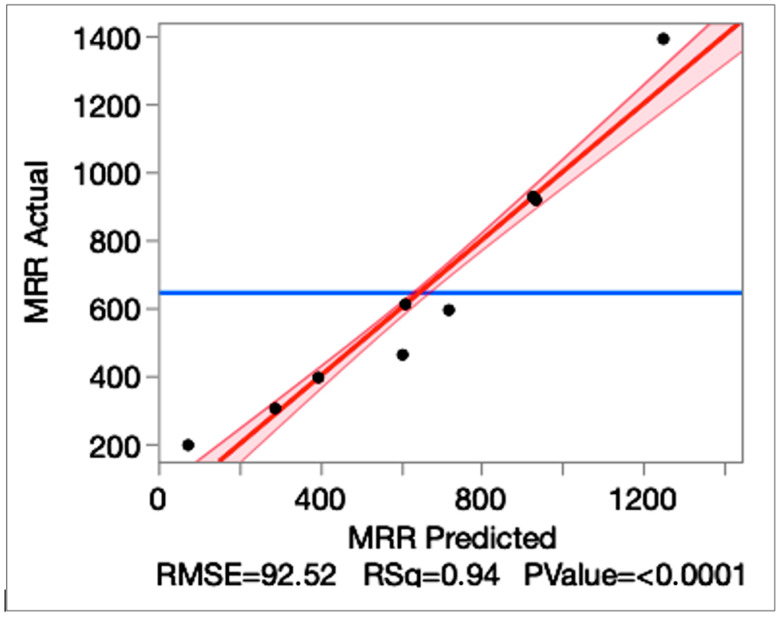
The MRR actual vs. MRR predicted plot graph.

The plot graph of the actual and predicted plot is given in Figure 6. It is observed that the plot graph exhibits a tight alignment of points along the 45° line (R^2^ = 0.94, Adj-R^2^ = 0.93, RMSE = 92.52 mm^3^/min; *p* < 0.0001). The situation confirms that the model explains over 93% of the observed variability in MRR and is suitable for optimization.

**Figure 7:**
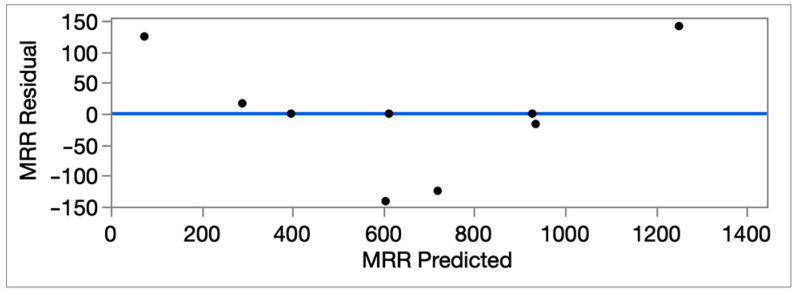
The MRR residual vs. MRR predicted plot graph.

The residuals vs. predicted plot is given in Figure 7. It is understood from Figure 7 that the regression model works reliably. The residuals are spread randomly around zero without any clear trend or pattern, which means the statistical assumptions of independence and constant variance are satisfied. To provide additional evidence, residual diagnostics are shown in Supplementary Figures S1 and S2. The Q–Q plot and histogram indicate that although the distribution is not perfectly normal, it remains approximately symmetric with stable variance, which supports the reliability of the regression model. The residual spread is approximately ±150 mm^3^/min, which corresponds to ~±10.8% of the full MRR scale (max predicted ≈ 1.391.7 mm^3^/min) with an RMSE value of 92.52 mm^3^/min (~6.6% of full scale; ~14.3% of the mean response). These outcomes indicate that the model’s predictions are stable and trustworthy across the experimental range.

**Figure 8:**
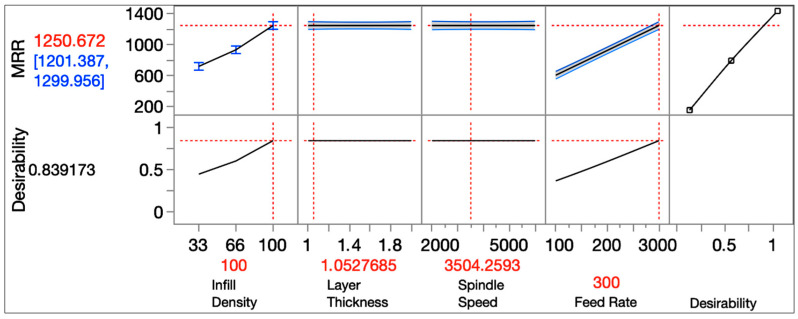
Desirability-based single-response optimization for MRR (The dotted lines represent the optimal points provided by the JMP software for desirability analysis, whereas the red values correspond to the associated parameter values of these dotted lines).

The desirability-based single-response optimization for MRR is given in Figure 8. The desirability value is found to be 0.839 at 100% infill, LT ≈ 1.053 mm, N ≈ 3504 rpm and F = 300 mm/min. With these settings, the model predicts MRR ≈ 1250.7 mm^3^/min with a 95% prediction interval of approximately 1201–1300 mm^3^/min. The profiler reaffirms the dominance of feed rate and infill density, indicating that desirability increases steadily with F, reaching its peak at ϕ = 100%. On the other hand, spindle speed and layer thickness exhibit nearly flat responses, consistent with their non-significance in the global regression analysis.

When optimization is made only for MRR, the model assigns intermediate values for spindle speed and layer thickness parameters because of their statistical non-significance (~3500 rpm spindle speed and ~1.05 mm layer thickness). This result is consistent with previous studies which state that in machining processes of FDM-printed parts, the MRR value depends on feed rate and material density [30]. However, when surface roughness (Ra) is included in the desirability function, the optimal spindle speed shifts to the maximum value (6000 rpm). This finding is also consistent with previous studies, which have documented the role of higher spindle speeds in reducing surface irregularities well [31,32]. Therefore, while spindle speed and layer thickness do not significantly influence MRR on their own, spindle speed becomes critical once surface quality is jointly optimized.

## 4. Conclusions

This study examines the CNC milling behavior of TPU 95A specimens with 100 mm × 25 mm × 6 mm dimensions and fabricated through the FDM process. Two responses are considered, which are material removal rate (MRR) and surface roughness (Ra). Input process parameters are spindle speed (2000, 4000 and 6000 rpm) and feed rate (100, 200 and 300 mm/min), together with infill density (33%, 66% and 100%) and layer thickness (1.0, 1.5 and 2.0 mm). To ensure realistic productivity values, the measured MRR values are corrected by multiplying them with a density-based correction factor and by including the non-cutting indexing (traverse) movements. These new values as MRR_corr_ are used for all MRR-related calculations and optimizations. Ra values can only be measured from specimens with 100% infill densities, as only these provided solid surfaces suitable for reliable surface characterization. A full factorial design consisting of 81 experiments is conducted to observe the influences of these parameters.

The analysis demonstrates that feed rate is the most effective parameter of MRR with statistical significance of *p* < 0.000, with MRR increasing from ~463 mm^3^/min at 100 mm/min feed to ~1392 mm^3^/min at 300 mm/min feed under 100% infill density. Infill density is observed to be the second most influential parameter. Specimens with 33% and 66% infill densities show a MRR reduction of −248.9 mm^3^/min and −33.3 mm^3^/min, respectively, compared to specimens with 100% infill density. This situation highlights the productivity advantage of solid specimens. The regression model achieves a high level of agreement with the experimental data (R^2^ = 0.94; Adj-R^2^ = 0.93), confirming its reliability for predicting MRR.

Analysis results for Ra reveal that Ra does not follow a linear path with respect to input process parameters; instead, the lowest surface roughness values are obtained only under specific parameter sets. The regression model for Ra shows a low level of agreement (R^2^ = 0.14). This situation indicates that the surface quality of flexible TPU 95A material is highly sensitive to parameter variations and not predictable by single-factor effects alone.

Optimization in this study is limited to a single-response MRR because the Ra model showed poor explanatory power (R^2^ = 0.14) and did not meet the assumptions for predictive use. Therefore, Ra for 100% infill is treated only descriptively to demonstrate milling benefits relative to as-printed surfaces (≈25–30 μm vs. best milled ≈13.8 μm), while MRR is modeled and optimized quantitatively across all infill densities.

In summary, the study highlights the results below:

Feed rate is the most dominant factor of MRR. Spindle speed and layer thickness have negligible effects on MRR.

Infill density has strong effects on both MRR and surface roughness. Maximum MRR values are obtained from specimens with 100% infill density, while surface roughness can only be measured from solid specimens.

Surface roughness in TPU 95A milling is highly sensitive to parameter changes, with spindle speed playing a decisive role in achieving the lower Ra values in the descriptive results, although Ra is excluded from optimization.

Overall, these findings can help to clarify the influence of machining parameters on the milling behavior of TPU parts produced by the FDM method. From a practical perspective, the findings of this study offer useful guidance for situations where FDM-printed TPU parts need additional finishing. In areas such as biomedical devices, sealing components, or flexible wearables, both accuracy and surface quality are critical. The results show that choosing higher feed rates, together with solid infill, can make machining more efficient, while milling itself consistently improves the roughness of as-printed surfaces. In practice, this means shorter finishing times, more predictable quality and easier integration of CNC milling into hybrid additive–subtractive workflows.

The novelty of this work is that it systematically examines the machinability of flexible TPU 95A by combining FDM and CNC milling in a single study. In doing so, it not only applies regression-based modeling of MRR but also provides a descriptive view of Ra, giving both methodological and application-oriented contributions to hybrid manufacturing.

Looking ahead, the approach used in this study can be extended to other polymers, machined by different methods such as turning or drilling. Also, supportive tests can be applied, such as strength or wear tests. These steps would broaden the scope and increase the practical relevance of the findings.

## Figures and Tables

**Table 1:** Mechanical properties of TPU 95A filaments.

Mechanical Properties	Value
Tensile Stress (at yield)	25.2 ± 2% MPa
Tensile Stress (at break)	8.1 ± 2% MPa
Tensile Modulus	37.6 ± 2% MPa
Elongation (at yield)	54% ± 2%
Elongation (at break)	570% ± 2%

**Table 2:** Technical specifications of the CNC vertical machining center.

Specifications	Unit	Value
Table dimensions	mm	400 × 700
Max. work piece weight	kg	300
(X*Y*Z) axis movement	mm	550 × 400 × 500
Spindle taper	d/d	BT*40/12.000
Transmission system	-	Direct drive
Cutting speed	m/min	1–12
(X*Y*Z) axis speed	m/min	36 × 36 × 30
Magazine capacity	-	24 tools
Spindle motor power	kW	5.5/7.5
Axis motors (X*Y*Z)	kW	1.5 × 2.0 × 3.0
Approx. weight	kg	3850

**Table 3:** Technical specifications of the end mill used for milling process.

Specifications	Unit	Value
Total length	mm	63
Tool diameter	mm	6
Shaft diameter	mm	6
Material type	-	Carbide
Flute number	-	4

**Table 4:** The factors and their corresponding levels used in experimental design.

Parameters	Unit	Levels of Factors		
		1	2	3
Infill Density (ϕ)	%	33	66	100
Layer Thickness (LT)	mm	1	1.5	2
Spindle Speed (N)	rpm	2000	4000	6000
Feed Rate (F)	mm/min	100	200	300

**Table 5:** Experimental conditions and measurement results.

Parameters				Results		
Infill Percentage (ϕ) %	Layer Thickness (LT) (mm)	Spindle Speed (N) (rpm)	Feed Rate (F) (mm/min)	MRR_nom_ (mm^3^/min)	MRR_corr_ (mm^3^/min)	Surface Roughness (Ra) (µm)
100	1	2000	100	600.00	463.92	26.65
			200	1200.00	927.84	13.82
			300	1800.00	1391.75	19.94
		4000	100	600.00	463.92	17.17
			200	1200.00	927.84	10.39
			300	1800.00	1391.75	15.25
		6000	100	600.00	463.92	14.33
			200	1200.00	927.84	16.96
			300	1800.00	1391.75	15.19
	1.5	2000	100	600.00	463.92	14.36
			200	1200.00	927.84	22.94
			300	1800.00	1391.75	10.71
		4000	100	600.00	463.92	17.42
			200	1200.00	927.84	17.13
			300	1800.00	1391.75	12.11
		6000	100	600.00	463.92	20.49
			200	1200.00	927.84	16.39
			300	1800.00	1391.75	15.3
	2	2000	100	600.00	463.92	13.75
			200	1200.00	927.84	17.66
			300	1800.00	1391.75	14.26
		4000	100	600.00	463.92	17.43
			200	1200.00	927.84	12.2
			300	1800.00	1391.75	15.02
		6000	100	600.00	463.92	15.85
			200	1200.00	927.84	20.53
			300	1800.00	1391.75	12.66
66	1	2000	100	396.00	306.19	-
			200	792.00	612.37	-
			300	1188.00	918.56	-
		4000	100	396.00	306.19	-
			200	792.00	612.37	-
			300	1188.00	918.56	-
		6000	100	396.00	306.19	-
			200	792.00	612.37	-
			300	1188.00	918.56	-
	1.5	2000	100	396.00	306.19	-
			200	792.00	612.37	-
			300	1188.00	918.56	-
		4000	100	396.00	306.19	-
			200	792.00	612.37	-
			300	1188.00	918.56	-
		6000	100	396.00	306.19	-
			200	792.00	612.37	-
			300	1188.00	918.56	-
	2	2000	100	396.00	306.19	-
			200	792.00	612.37	-
			300	1188.00	918.56	-
		4000	100	396.00	306.19	-
			200	792.00	612.37	-
			300	1188.00	918.56	-
		6000	100	396.00	306.19	-
			200	792.00	612.37	-
			300	1188.00	918.56	-
33	1	2000	100	198.00	153.10	-
			200	396.00	306.19	-
			300	594.00	459.28	-
		4000	100	198.00	153.10	-
			200	396.00	306.19	-
			300	594.00	459.28	-
		6000	100	198.00	153.10	-
			200	396.00	306.19	-
			300	594.00	459.28	-
	1.5	2000	100	198.00	153.10	-
			200	396.00	306.19	-
			300	594.00	459.28	-
		4000	100	198.00	153.10	-
			200	396.00	306.19	-
			300	594.00	459.28	-
		6000	100	198.00	153.10	-
			200	396.00	306.19	-
			300	594.00	459.28	-
	2	2000	100	198.00	153.10	-
			200	396.00	306.19	-
			300	594.00	459.28	-
		4000	100	198.00	153.10	-
			200	396.00	306.19	-
			300	594.00	459.28	-
		6000	100	198.00	153.10	-
			200	396.00	306.19	-
			300	594.00	459.28	-

**Table 6:** Model summary statistics for 100% infill density.

Statistics	Value	
	MRR	Ra *
R^2^	1.00	0.14
Adjusted R^2^	1.00	0.03
RMSE (mm^3^/min)	0.0026	3.62
Mean of Response (mm^3^/min)	927.84	16.15
Observations	27	27

* Ra statistics are reported for completeness but excluded from optimization due to weak model fit.

**Table 7:** ANOVA results for 100% infill density.

Source	DF	Sum of Squares		Mean Square		F-Ratio		Prob > F	
	MRR and Ra	MRR	Ra	MRR	Ra	MRR	Ra	MRR	Ra
Model	3	3,873,908.3	48.74	1,291,303	16.25	1.98 × 10^11^	1.25	<0.0001 *	0.32
Error	23	0.00015	300.22	6.522 × 10^−6^	13.06				
C. Total	26	3,873,908.3	348.96						

* Significant at α = 0.05.

**Table 8:** Parameter estimates for 100% infill density.

Source	Estimate		Std Error		t-Ratio		Prob > |t|	
	MRR	Ra	MRR	Ra	MRR	Ra	MRR	Ra
Intercept	0.0067	21.58	0.0026	3.58	2.64	6.03	0.0148	<0.0001 *
LT	0	−1.15	0.0012	1.70	0.00	−0.67	1.0000	0.51
N	0	0.0002	3.01 × 10^−7^	4.26 × 10^−4^	0.00	−0.42	1.0000	0.69
F	4.64	−0.016	6.019 × 10^−6^	0.009	770,712	−1.76	<0.0001 *	0.09

* Significant at α = 0.05.

**Table 9:** Effect test results for 100% infill density.

Source	Nparm	DF	Sum of Squares		F-Ratio		Prob > F	
	MRR and Ra	MRR and Ra	MRR	Ra	MRR	Ra	MRR	Ra
LT	1	1	0.00	5.94	0.00	0.46	1.00	0.51
N	1	1	0.00	2.27	0.00	0.18	1.00	0.69
F	1	1	3,873,908.3	40.54	5.94 × 10^11^	3.11	<0.0001 *	0.09

* Significant at α = 0.05.

**Table 10:** Model summary statistics for all infill densities.

Statistics	Value
	MRR
R^2^	0.94
Adjusted R^2^	0.93
RMSE (mm^3^/min)	92.52
Mean of Response (mm^3^/min)	645.67
Observations	81

**Table 11:** ANOVA results for MRR.

Source	DF	Sum of Squares	Mean Square	F-Ratio	Prob > F
Model	5	9,479,822	1,895,964	221.51	<0.0001 *
Error	75	641,945	8559		
C. Total	80	10,121,767			

* Significant at α = 0.05.

**Table 12:** Parameter estimates for MRR.

Source	Estimate	Std Error	t-Ratio	Prob > |t|
Intercept	0.002	52.92	0.00	1.00
Infill Density (33%)	−248.87	14.54	−17.12	<0.0001 *
Infill Density (66%)	−33.30	14.54	−2.29	0.0248 *
LT	1.474 × 10^−14^	25.17975	0.00	1.00
N	8.084 × 10^−19^	0.006	0.00	1.00
F	3.23	0.13	25.64	<0.0001 *

* Significant at α = 0.05.

**Table 13:** Effect test results for MRR.

Source	Nparm	DF	Sum of Squares	F-Ratio	Prob > F
ϕ	2	2	3,851,810.7	225.0082	<0.0001 *
LT	1	1	2.932 × 10^−27^	0.0000	1.0000
N	1	1	1.4117 × 10^−28^	0.0000	1.0000
F	1	1	5,628,011.6	657.5343	<0.0001 *

* Significant at α = 0.05.

## Data Availability

The original contributions presented in the study are included in the article and further inquiries can be directed to the corresponding author.

## Supplementary Materials

The following supporting information can be downloaded at: https://www.mdpi.com/article/10.3390/mi16101078/s1, Supplementary Figure S1: Q–Q plot of residuals showing approximate symmetry; Supplementary Figure S2: Histogram of residuals.
