# Supplementary figures and images for: Regression Modeling and Optimization of CNC Milling Parameters for FDM-Printed TPU 95A Components
Source: Micromachines (Basel). 2025 Sep 24;16(10):1078. doi: 10.3390/mi16101078 (PMC12566470; doi:10.3390/mi16101078)

Q-Q Plot of Residuals

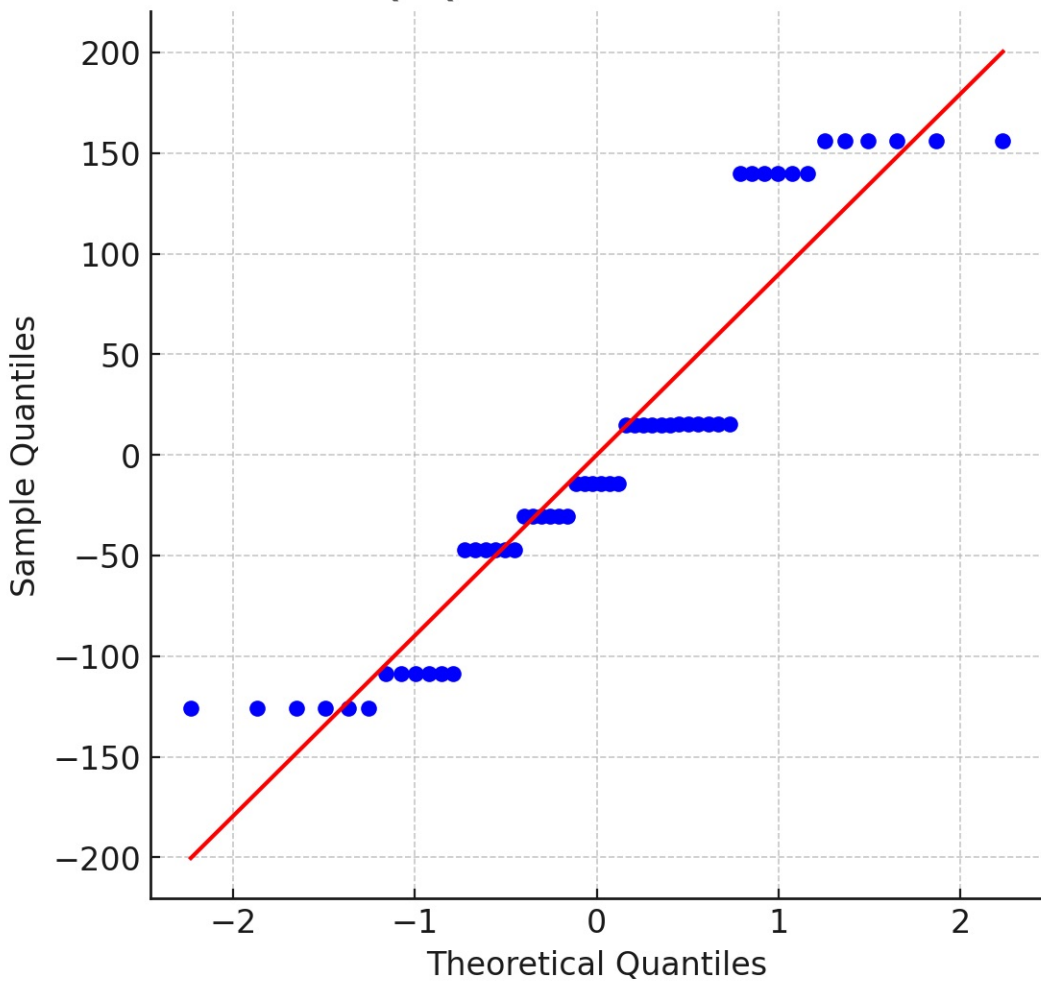

Supplement: Supplementary file 1 [file micromachines-16-01078-s001.zip › Fgure S1.pdf]

# Histogram of Residuals

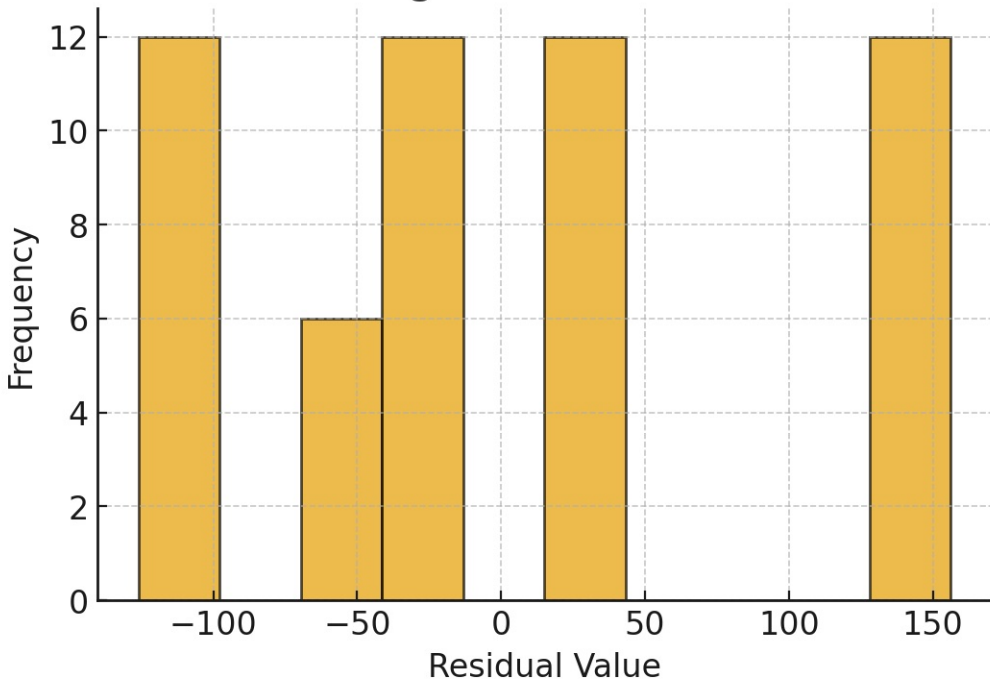

Supplement: Supplementary file 1 [file micromachines-16-01078-s001.zip › Fgure S2.pdf]
